# Klassevirus Infection in Children, South Korea

**DOI:** 10.3201/eid1610.100539

**Published:** 2010-10

**Authors:** Tae-Hee Han, Cheol-Hwan Kim, Ju-Young Chung, Sang-Hun Park, Eung-Soo Hwang

**Affiliations:** Author affiliations: Inje University College of Medicine, Seoul, South Korea (T.-H. Han, J.-Y. Chung); Sungkyunkwan University, Seoul (C.-H. Kim);; Seoul Metropolitan Government Research Institute of Public Health and Environment, Seoul (S.-H. Park);; Seoul National University College of Medicine, Seoul (E.-S. Hwang)

**Keywords:** Kobuvirus, Picornaviruses, children, gastroenteritis, enteric infections, viruses, Korea, dispatch

## Abstract

To investigate prevalence and clinical characteristics of klassevirus in South Korea, we performed molecular screening in fecal and nasopharyngeal samples from hospitalized children with gastroenteritis. A total of 26 (8.8%) of 294 fecal samples were positive for klassevirus. Klassevirus may be a possible cause of gastroenteritis.

Identification of new picornaviruses (family *Picornaviridae*) in fecal samples has increased because of new molecular methods (*1**,**2*), but clinical significance is not clear. The genus *Kobuvirus* belongs to the family *Picornaviridae* and contains 3 species: *Aichi virus*, *Bovine kobuvirus*, and *Porcine kobuvirus*. Aichi virus was first identified as an etiologic agent of gastroenteritis, but the connection has not yet been proven (*3*).

In 2009, Holtz et al. (*4*) identified a new picornavirus, kobu-like virus (klassevirus), associated with feces and sewage, in a fecal sample from a child from Australia; the complete genome of this virus has been reported (*5*). However, because the prevalence of klassevirus-1 and its clinical role in gastroenteritis remain unclear, we investigated its prevalence and clinical characteristics in South Korea.

## The Study

We analyzed 3 groups of samples. The first group (retrospective fecal group) comprised archived virus-negative fecal samples from 342 children <6 years of age hospitalized with gastroenteritis at Sanggyepaik Hospital during September 2007–April 2009 (*6**,**7*). The second group (prospective group) comprised 294 fecal samples prospectively collected during May 2009–February 2010 from hospitalized children <17 years of age at Sanggyepaik Hospital who had gastroenteritis. The third group (nasopharyngeal aspirate group) comprised 142 archived virus-negative nasopharyngeal aspirates from hospitalized children <6 years of age who had acute lower respiratory tract infections during September 2006–June 2007 (*8*). The ethics committee of Sanggyepaik Hospital, Inje University, approved the study protocol.

All fecal samples were tested for common bacterial diarrheal pathogens by routine microbiologic methods, as described (*9*). Rotavirus and adenoviruses 40 and 41 were identified by using the ELISA kits Rotaclone and Adenoclone 40/41 (Meridian Bioscience, Cincinnati, OH, USA). The seminested reverse transcription–PCR (RT-PCR) for norovirus, which used primers based on the capsid region and for human astrovirus based on open reading frame 1a were performed as described (*10**,**11*). PCR for human bocavirus 2, which used primers based on the nonstructured gene, and nested RT-PCR for Saffold virus (SAFV), which used primers based on the 5′ noncoding region, were performed as described (*1**,**6*). To detect Aichi virus, we conducted a nested PCR with primer sets based on the 3CD junction region, as described (*12**,**13*).

To detect klassevirus, we performed the first reactions of 2 nested RT-PCRs by using the following primers: LG0118 and LG0117 for 3D region and LG0119 and LG0136 for viral protein (VP) 0/VP1 gene, as described (*4*) (Table). The second reactions of each RT-PCR were conducted by using newly designed primers based on the Klasse mel1 sequence (GQ253936): KL3DF and KL3DR for the 3D region and KLVPF and KLVPR for the VP0/VP1 region at 40 cycles of 94°C for 30 s, 55°C for 30 s, and 72°C for 60 s. A third RT-PCR for the 2C region of klassevirus amplifying a 345-bp fragment was performed by modified primers KL2C-F1 and KL2C-R1 for the first reaction and KL2C-F2 and KL2C-R2 for the second reaction under the following conditions: 40 cycles of 94°C for 30 s, 57°C for 30 s, and 72°C for 60 s. Amplicon was purified by using QIAquick (QIAGEN, Valencia, CA, USA) and sequenced in both directions with the BigDye Terminator version 3.1 Cycle Sequencing kit (Applied Biosystems, Foster City, CA, USA). Nucleotide sequences were aligned by using BioEdit version 7.0 and presented in a phylogenic tree prepared in MEGA 4.1 (www.megasoftware.net). The tree was constructed by using the neighbor-joining method with Kimura 2-parameter estimation.

**Table:** Primers used in PCR for Aichi virus and klassevirus*

Virus/primer	Sequence (5′ → 3′)	Gene	Reference
Aichi virus			
6261	ACA CTCCCACCTCCCGCCAGTA (1st)	3CD	(*12*)
6779	GGAAGAGCTGGGTGTCAAGA		
C94b	GACTTCCCCGGAGTCGTCGTCT (2nd)	3CD	(*13*)
246k	GACATCCGGTTGACGTTGAC		
Klassevirus			
LG0118	ATGGCAACCCTGTCCCTG AG (1st)	3D	(*4*)
LG0117	GAAACCCAACCACGCTGTA		(*4*)
KL3DF	GTCTGGTCTATYGAYTACTCTTGCTTT (2nd)	3D	This study
KL3DR	AGGACGGAGTAGGGRGTRAA		This study
LG0119	GCTAACTCTAATGCTGCCACC (1st)	VP0/VP1	(*4*)
LG0136	GCTAGGTCAGTGGAAGGATCA		(*4*)
KLVPF	GTCACYCCMAACACCTCCACTGAAG (2nd)	VP0/VP1	This study
KLVPR	TTCTGCRCCATCRGCTCCCGA		This study
KL2C-F1	CTCGCYGAGGACATCACGGA (1st)	2C	This study
KL2C-R1	GTACAGGTACACRACCAGTGGCT		This study
KL2C-F2	AATCTGCTGCCCAGGCCGC (2nd)	2C	This study
KL2CR2	AGGGAGATGGCRGAGAGAGCTGT		This study

*VP, viral protein.

Among 342 children in the retrospective fecal group, 15 (4%) samples were positive for klassevirus by RT-PCR. All samples were positive by 3 kinds of RT-PCR for the 3D, VP0/VP1, and 2C regions. Klassevirus-positive samples in this group were frequently found in February 2009 (33%) and March 2009 (20%)

We tested 294 fecal samples collected prospectively for klassevirus RNA. Ages of children in the study were 109 (37%) <12 months of age; 159 (54%) 12–60 months of age; and 26 (9%) >60 months of age (median 18 months, range 1–174 months). The male:female ratio was 1.1:1 (155:139). Enteropathogenic bacteria were found in 6 (2.0%) children, *Salmonella* spp. in 4 (1.0%), and enterotoxigenic *Escherichia coli* in 2 (0.6%). Rotavirus (13.7%) and norovirus (10.6%) were the most prevalent viral agents. Adenovirus, human bocavirus 2, sapovirus, and human bocavirus 1 were detected in 6 (2%), 3 (1%), 2 (0.6%), and 2 (0.6%) children, respectively. SAFV was detected in 1 child, and the virus belonged to SAFV-1 sublineage. Astrovirus and Aichi virus were not found.

Klassevirus was detected in 11 (4%) children in the prospective group, most frequently in June and August 2009 (27% and 36% of detections, respectively). All samples were positive by RT-PCR for the 3D and 2C regions, but the VP0/VP1 region could be amplified in only 8 samples, possibly because of the variance of the strains and sensitivity of the primer sets. Other viral agents were co-detected in 3 (12%). In 142 virus-negative nasopharyngeal aspirates, klassevirus was not detected.

The 26 klassevirus-positive patients ranged in age from 2 months to 175 months (median 31 months, mean 51 months). All klassevirus-positive patients had diarrhea; other symptoms manifested were fever, vomiting, cough, rhinorrhea, and skin rash. No patients had underlying medical problems, and all recovered completely.

**Figure A1:**
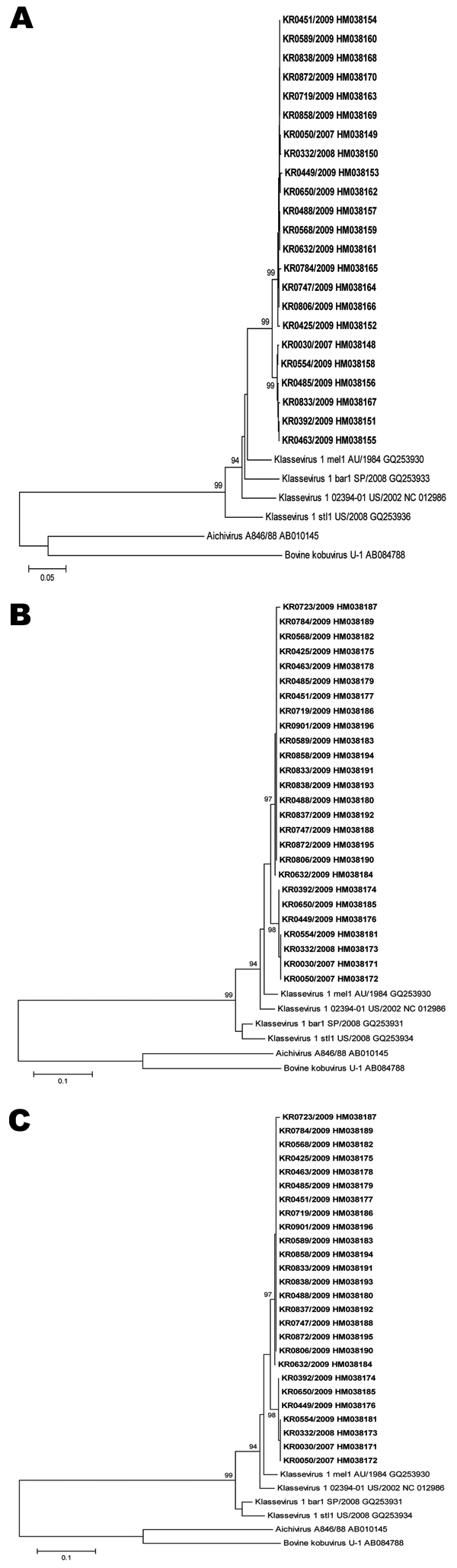
Phylogenetic analysis of the A) partial viral protein (VP) 0/VP1 (852 bp), B) 3D (668 bp), and C) 2C (345 bp) gene sequences of klassevirus-1 strains. The klassevirus-1 strains isolated in this study are indicated in **boldface**. The tree was constructed by using the neighbor-joining method with Kimura 2-parameter estimation. The bootstrap values from 1,000 replicates are present on each branch. The nucleotide sequences identified in this study have been deposited in GenBank under accession nos. HM38122–HM038147 (3D gene), HM038148–HM038170 (VP0/VP1 gene), and HM038171–HM038196 (2C gene). AU, Australia; KR, Korea; SP, Spain; US, United States.

Phylogenetic analysis showed that Korean isolates in this study clustered into reference strains, KV US/2002 (NC 012986) and KV AU/1984 (GQ 253930) (Figure A1), but the sequence variation was limited. The ranges of nucleotide differences and amino acid differences between Korean strains and reference strains were 5%–11% (Figure A1) and 0%–14% (data not shown), respectively.

## Conclusions

We found a higher prevalence of klassevirus in feces from children with gastroenteritis than has been found in previous studies (*4**,**5*), possibly because of the study population and different primers. Most (76.9%) klassevirus-positive children were <3 years of age, although the virus was detected in children >6 years of age. Co-infection of klassevirus with other viral agents was lower than co-infection with recently identified viruses, such as SAFV and cosavirus (*1**,**2*). These results suggest that klassevirus could be a possible cause of gastroenteritis in children; however, further studies that include asymptomatic control groups are needed to exclude the possibility of klassevirus being an innocent bystander. Recently, Li et al. (*14*) reported that salivirus, which has 90% nt similarity with klassevirus, might have a role in gastroenteritis. However, this virus could not be differentiated from klassevirus in the phylogenetic analyses based on 3 different regions (data not shown).

We did not detect klassevirus in nasopharyngeal aspirates despite respiratory symptoms in 26.9% of klassevirus-positive children. These results indicate that klassevirus might not be an etiologic agent of acute lower respiratory tract infections; additional studies are required to be conclusive. We detected SAFV in 1 patient; a recent study shows high prevalence of this virus in healthy children and in children with gastroenteritis (*15*), perhaps because of different assay sensitivities. In this study, the results of phylogenetic assay of the 3 different regions showed low genetic diversity of klassevirus, which suggest an outbreak by a single circulating klassevirus strain. A definite seasonality of klassevirus infection was not observed. In conclusion, we have detected klassevirus in children with gastroenteritis, which suggests a possible association between this virus and gastroenteritis.
